# Towards the Oral Treatment of Ileo-Colonic Inflammatory Bowel Disease with Infliximab Tablets: Development and Validation of the Production Process

**DOI:** 10.3390/pharmaceutics11090428

**Published:** 2019-08-23

**Authors:** Bahez Gareb, Silke Posthumus, Max Beugeling, Pauline Koopmans, Daan J. Touw, Gerard Dijkstra, Jos G.W. Kosterink, Henderik W. Frijlink

**Affiliations:** 1 Department of Clinical Pharmacy and Pharmacology, University Medical Center Groningen, University of Groningen, Hanzeplein 1, 9713 GZ Groningen, The Netherlands; 2 Department of Pharmaceutical Technology and Biopharmacy, Groningen Research Institute of Pharmacy, University of Groningen, Antonius Deusinglaan 1, 9713 AV Groningen, The Netherlands; 3 Department of Pharmaceutical Analysis, Groningen Research Institute of Pharmacy, University of Groningen, Antonius Deusinglaan 1, 9713 AV Groningen, The Netherlands; 4 Department of Gastroenterology and Hepatology, University Medical Center Groningen, University of Groningen, Hanzeplein 1, 9713 GZ Groningen, The Netherlands; 5 Department of PharmacoTherapy, -Epidemiology and -Economics, Groningen Research Institute of Pharmacy, University of Groningen, Antonius Deusinglaan 1, 9713 AV Groningen, The Netherlands

**Keywords:** ColoPulse, infliximab, drug targeting, topical, ileo-colonic, inflammatory bowel disease

## Abstract

Infliximab (IFX) is an intravenously administered monoclonal antibody antagonizing the effects of tumor necrosis factor-alpha (TNF) systemically and is efficacious in the treatment of inflammatory bowel disease (IBD). However, studies suggest that the anti-inflammatory effects result from local immunomodulation in the inflamed regions. Furthermore, topical inhibition of TNF in IBD ameliorates inflammation. We therefore hypothesized that orally administered IFX targeted to the ileo-colonic region in IBD may be an efficacious new treatment option. This study describes the development and validation of the production process of ileo-colonic-targeted 5 mg IFX tablets (ColoPulse-IFX) intended for the oral treatment of IBD by means of producing three consecutive validation batches (VAL1, VAL2, and VAL3, respectively). UV-VIS spectroscopy, HPLC-SEC analysis (content, fragments, aggregates), fluorescence spectroscopy (tertiary protein structure), and ELISA (potency) showed no noticeable deviations of IFX compounded to ColoPulse-IFX compared to fresh IFX stock. The average ± SD (*n* = 10) IFX content of VAL1, VAL2, and VAL3 was 96 ± 2%, 97 ± 3%, and 96 ± 2%, respectively, and complied with the European Pharmacopeia (Ph. Eur.) requirements for Content Uniformity. The average ± SD (*n* = 3) ColoPulse-IFX potency was 105 ± 4%, 96 ± 4%, and 97 ± 5%, respectively, compared to fresh IFX stock. The IFX release profile from the tablet core was complete (≥85%) after 10 min in simulated ileum medium. The in vitro coating performance of ColoPulse-IFX showed that the formulation was targeted to the simulated ileo-colonic region. Stability data showed that ColoPulse-IFX was stable for up to 6 months stored at 25 °C/60% RH. Based on these results, the production process can be considered validated and its application is discussed in light of the rationale and available evidence for the topical treatment of IBD with IFX.

## 1. Introduction

Ulcerative colitis (UC) and Crohn’s disease (CD) are inflammatory bowel diseases (IBD) affecting the gastrointestinal tract (GIT). Both diseases are characterized by their relapsing behavior, chronic mucosal inflammation, and frequent hospitalization, resulting in a marked decrease in quality of life. In UC, the inflammation is limited to the colonic mucosa whereas the inflammation in CD is transmural and can affect the entire GIT. In approximately 50% of IBD patients, the inflammation affects the terminal ileum and colon [1,2,3,4,5].

The exact pathogenesis of IBD is unclear, although research shows a major role of genetic and environmental factors contributing to epithelial barrier dysfunction. As a consequence, increased exposure of the intestinal wall to luminal antigens, such as microbiota and xenobiotics, induce an acute immune response driven by the innate immune cells. This immune response leads to tissue injury due to the secreted reactive oxygen species and pro-inflammatory mediators such as interleukin (IL)-1β, IL-6, and tumor necrosis factor-alpha (TNF). The activation of the adaptive immune cells by the pro-inflammatory cytokines perpetuate the inflammatory state, resulting in chronic inflammation [6,7,8,9].

TNF is a pleiotropic cytokine involved in the regulation of cell proliferation, survival, and death. It is produced as a transmembrane protein (tmTNF) and can be enzymatically cleaved to yield soluble TNF (sTNF). Both forms are active and share similar as well as distinctive effects. TNF aids in the host defense against infections and local injury. In IBD however, the elevated levels in the intestinal mucosa and lamina propria are associated with the activation of pro-inflammatory immune cells, angiogenesis, impaired barrier function, and suppression of regulatory immune cells, which could attenuate inflammation [8,10,11,12].

Infliximab (IFX) is a chimeric monoclonal antibody against TNF and therefore antagonizes the TNF-induced effects. In moderate-to-severe UC refractory to corticosteroid and immunosuppressive drugs, IFX induces clinical remission and mucosal healing [13,14,15,16]. Furthermore, IFX is efficacious in the induction and maintenance of clinical remission as well as mucosal healing in CD [15,17,18,19]. IFX is administered intravenously since it is a monoclonal antibody and thus antagonizes TNF systemically. This has been associated with adverse effects, such as an increased risk of infection [20,21]. In addition, systemic exposure may induce the formation of antibodies to infliximab (ATI), which is associated with loss of response to IFX and infusion reactions [22,23,24].

Interestingly, studies show that the marked anti-inflammatory effects of IFX in IBD are resulting from local immunomodulation in the ileo-colonic region [25,26,27,28]. Additionally, data suggest that IFX concentrations in these inflamed tissues correlate with a clinical response instead of serum IFX concentrations alone. This may partly explain therapy failure despite therapeutic serum concentration [29,30]. Moreover, studies show that local inhibition of TNF in the inflamed regions of CD as well as UC patients ameliorates inflammation and induces a favorable response [31,32,33,34,35]. These observations suggest that topically delivered IFX may be efficacious in the treatment of IBD by inducing a localized effect. Major challenges in accomplishing topical delivery of IFX in ileo-colonic IBD are targeting IFX to the site of inflammation in a suitable dosage form and the stability of IFX thereafter in the intestinal lumen.

The ColoPulse coating technology is specifically developed to target the ileo-colonic region in humans after oral administration. The coating consists of a pH sensitive polymer (pH threshold ≥ 7) in which the super-disintegrant sodium croscarmellose is incorporated in the coating matrix. The intraluminal pH of the GIT rises to 7.5 for a short period of time (30 min) during transit from the jejunum to the ileum. Thereafter, the pH drops to 6 in the colon [36,37,38,39]. The ColoPulse coating utilizes this short period of pH rise in the ileum before reaching the colon. It resists the pH of the stomach, duodenum, and jejunum. However, when the pH rises to ≥7 in the terminal ileum, coating disintegration starts. Subsequently, water uptake by the super-disintegrant incorporated in the coating matrix swells rapidly, inducing fast coating disintegration, even though the pH rises for a short period of time (30 min) to ≥7.

We have shown in five clinical trials that oral dosage forms coated with the ColoPulse coating can be targeted to the ileo-colonic region in healthy subjects as well as CD patients [38,40,41,42,43]. Moreover, in a preliminary study we have shown that the production of ColoPulse-coated IFX tablets is feasible and that the stresses associated with tablet production and coating were not detrimental to the protein structure [44]. The in vitro stability of IFX in simulated gastrointestinal (GI) systems as well as in vivo stability of antibodies in the GIT is encouraging [45,46,47,48,49]. Therefore, we hypothesized that orally administered, ileo-colonic-targeted IFX tablets may be efficacious in the treatment of ileo-colonic IBD.

The objective of this study was to develop and validate the production process of ileo-colonic-targeted 5 mg IFX tablets in view of the oral treatment of ileo-colonic IBD (Table 1). The validation was performed by producing three consecutive batches and analyzing the tablets for IFX content, structural integrity, and potency as well as ileo-colonic targeting in an in vitro gastrointestinal simulation system (GISS). The stability of the novel formulation was also investigated for six months stored at 25 °C/60% RH.

## 2. Materials and Methods

### 2.1. Chemicals

Infliximab (Remsima, Celltrion, Incheon, Korea), methacrylic acid–methyl methacrylate copolymer 1:2 (Eudragit S100, Evonik, Essen, Germany), polysorbate 20, dextran 70 kDa, trehalose (Sigma-Aldrich, St. Louis, MO, USA), polyethylene glycol 6000 (PEG 6000, Fagron, Capelle aan den IJssel, The Netherlands), sodium stearyl fumarate (JRS Pharma, Rosenberg, Germany), acetone, sodium hydroxide, hydrochloric acid 37% (VWR, Fontenay-sous-Bois, France), talc, potassium dihydrogen phosphate, sodium chloride (Spruyt-Hillen, IJsselstein, The Netherlands), croscarmellose sodium (FMC, Brussels, Belgium), sodium dihydrogen phosphate dihydrate, disodium hydrogen phosphate dihydrate (Merck, Darmstadt, Germany), microcrystalline cellulose (DMV Fonterra Excipients, Foxhol, The Netherlands), and Inulin (Frutafit TEX!, Sensus, Roosendaal, The Netherlands) were all used as received from their respective suppliers.

### 2.2. Study Design

IFX is a protein, and as such, compounding it to tablets may be detrimental to the protein structure and therefore its potency to antagonize TNF. Therefore, IFX was reconstituted in a sugar solution and subsequently lyophilized to yield IFX-sugar glass powder, which was used for further compounding. Incorporating proteins in a sugar glass matrix stabilizes proteins [50]. The lyophilized cake in the unprocessed Remsima vials already contained the sugar sucrose, which is a disaccharide with a glass transition temperature (*T*_g_) of 77 °C [51]. It was expected that compounding—most importantly, the coating procedure, since the coating contains an acetone–water mixture—would be detrimental to the IFX structure and stability due to this relatively low *T*_g_, the solubility of sucrose in water (high solubility) and acetone (low solubility), and the potential of water and acetone to act as a plasticizer on the IFX-sucrose matrix [52]. In view of increasing the stabilizing effects of the sugar glass matrix in which IFX was suspended, three different sugar solutions based on the *T*_g_, size, and molecular flexibility were chosen for the reconstitution of the powder in the Remsima vials, yielding a 1:1 ratio of the already present sucrose and the added sugars. This solution was subsequently lyophilized. The three different sugars used were inulin (*T*_g_ = 156 °C, oligosaccharide, molecularly flexible, [IFX-I]), dextran 70 kDa (*T*_g_ = 224 °C, polysaccharide, molecularly inflexible, [IFX-D]), or trehalose (*T*_g_ = 122 °C, disaccharide, molecularly flexible [IFX-T]) [53]. Subsequently, biconvex 9 mm (intended tablet size) compacts produced at different compaction forces of the lyophilized IFX-sugar glass were made to investigate whether IFX could be released from the compacted matrix without the addition of tablet excipients. Based on this experiment, the optimal sugar solution for the reconstitution of the Remsima powder was chosen for the production of the IFX tablets.

Three consecutive validation batches (VAL1, VAL2, and VAL3, respectively) of lyophilized IFX-sugar glass powder containing the optimal sugar were produced for further investigation. This powder was first analyzed for IFX content. Thereafter, uncoated tablets were produced (Uncoated-IFX) and analyzed for content. Subsequently, ColoPulse-coated IFX tablets (ColoPulse-IFX) were produced and analyzed for content, protein structure, and potency. ColoPulse-coated IFX tablets containing 25 mg caffeine in the tablet core (ColoPulse-IFX-caff) were produced as well to investigate the in vitro coating performance with UV-VIS in the GISS. This was done since the IFX release profile could not be analyzed accurately with UV-VIS or HPLC-SEC due to the resulting low concentration of IFX in the GISS (see also Section 2.13 and Section 2.14). Finally, the stability of the formulation was investigated for 6 months stored at room temperature (25 °C/60% RH) according to the ICH guideline [54]. Table 2 shows the components of all the produced formulations.

### 2.3. Lyophilization

The powder in Remsima vials containing 100 mg IFX was reconstituted in a solution of 10 mL 5% (*w*/*v*) sugar (inulin, dextran 70 kDa, or trehalose) dissolved in demineralized water. This solution was aliquoted in lyophilization vials, snap-frozen using liquid nitrogen, and then lyophilized (Christ, Salm & Kipp, Breukelen, The Netherlands). The primary drying step consisted of 24 h at −35 °C at 0.220 mbar followed by the secondary drying step consisting of 30 h at 25 °C at 0.050 mbar. The pressure and temperature were gradually adjusted until the targets were reached. Thereafter, the lyophilized cake was sieved (0.4 mm), resulting in the IFX-sugar glass powder (IFX-I, IFX-D, and IFX-T, respectively).

### 2.4. IFX-Sugar Glass Compacts

IFX-I, IFX-D, and IFX-T, respectively, were compacted at 1 kN and 3 kN with a rate of 0.5 kN/s (Instron, Norwood, MA, USA). The compacts weighed 100 mg and were biconvex in shape and 9 mm in diameter (intended tablet size). These compacts were gently pulverized in a mortar with a pestle, quantitatively transferred to a 50 mL tube, and 10 mL PBS pH 6.8 (150 mM) containing 0.05% (*w*/*v*) polysorbate 20 was added. These samples were mixed for 60 min and thereafter filtered (0.45 µm). The IFX content of the filtrate was analyzed with UV-VIS spectroscopy at λ = 280 nm (Thermo Fischer Scientific, Madison, WI, USA). Non-compacted, with mortar and pestle pulverized IFX-sugar glass powder was analyzed in the same manner to investigate whether the pulverization of the IFX-sugar glass compacts was detrimental to the IFX content.

### 2.5. Tablet Core Production

Tablet components were mixed manually (Table 2). All excipients, with the exception of the lubricant sodium stearyl fumarate, were mixed thoroughly until a homogenously mixture resulted. Subsequently, the lubricant was added and mixed roughly throughout the powder mixture. This mixture was compacted at 3 kN with a rate of 0.5 kN/s (Instron, Norwood, MA, USA).

### 2.6. Tablet Coating

Uncoated-IFX tablets were coated with the ColoPulse coating. The coating suspension consisted of Eudragit S100/PEG 6000/croscarmellose sodium/talc at a ratio of 7/1/3/2 (*w*/*w*) in a solvent mixture of acetone/water 97/3 (*v*/*v*). This coating suspension was continuously sprayed onto the tablets in a mini-rotating drum equipped with a hot air blower for the evaporation of the solvent mixture and to induce coating film formation.

### 2.7. Resistance to Crushing and Friability Tests

Resistance to crushing of the uncoated and coated tablets was determined with a tablet hardness tester (Erweka, Heusenstamm, Germany). The friability of 19 Uncoated-IFX (mass of 6.7 g) and 16 ColoPulse-IFX (mass of 6.8 g) tablets per experiment was determined according to the European Pharmacopoeia (Ph. Eur.) in a tablet friability apparatus (Erweka, Heusenstamm, Germany) [55].

### 2.8. Sample Preparation

Uncoated or coated tablets (Table 2) were gently pulverized in a mortar with pestle, quantitatively transferred to a 50 mL tube, and 10 mL PBS pH 6.8 (150 mM) containing 0.05% (*w*/*v*) polysorbate 20 was added. This suspension was mixed for 15 min after which the supernatant was filtered through a 0.45 µm filter. Samples for the IFX-sugar glass powder were prepared in the same manner without pulverization since this was already a powder. The filtered samples were analyzed with UV-VIS, HPLC-SEC, fluorescence spectroscopy, and ELISA. For the stress tests, filtered samples were stored for 1 h at 60 °C and analyzed with the same techniques.

### 2.9. UV-VIS Spectroscopy Analysis

UV-VIS spectroscopy was applied to investigate protein structure abnormalities due to compounding that could be visible in the spectrum. The UV-VIS spectrum of filtered samples was analyzed from λ = 190–400 nm (Thermo Fischer Scientific, Madison, WI, USA). Special attention was paid to potential deviations in the UV-VIS spectrum of ColoPulse-IFX compared to the UV-VIS spectrum of ColoPulse-placebo spiked with 5 mg fresh IFX stock. UV-VIS analysis was deemed inappropriate for IFX content analysis since the tablet excipients, predominantly Eudragit S100 from the ColoPulse coating, interfered with the content analysis at λ = 280 nm.

### 2.10. HPLC-SEC Analysis

Filtered samples were analyzed with HPLC-SEC to analyze the IFX content as well as to investigate whether potentially formed aggregates, fragments, or degradation products were formed due to compounding. HPLC (Dionex, Germering, Germany) was coupled with a SEC column (Phenomenex, BioSep 5 µm SEC-s3000 290 Å, Torrance, CA, USA). The wavelength, injection volume, flow rate, column temperature, run time, and mobile phase were λ = 280 nm, 20 µL, 1.0 mL/min, 22 °C, 15 min, and PBS pH 6.8 (150 mM) containing 0.05% (*w*/*v*) polysorbate 20, respectively.

Content Uniformity of ColoPulse-IFX was tested according to the Ph. Eur. by analyzing the content of 10 individual ColoPulse-IFX tablets and calculating the acceptance value according to the Ph. Eur. monograph *Consistency of Formulated Preparations*. The requirement to comply with the test was an acceptance value of ≤15.0 [56].

### 2.11. Fluorescence Spectroscopy Analysis

Fluorescence spectroscopy was applied to investigate the tertiary protein structure of IFX and potential deviations thereof due to compounding [57]. Filtered samples were further diluted 10× in PBS pH 6.8 (150 mM) containing 0.05% (*w*/*v*) polysorbate 20. This sample was transferred to a 10.00 mm fluorescence quartz cuvette. The sample in the secured quartz cuvette was stirred magnetically to prevent photobleaching. The intrinsic fluorescence of the sample was analyzed with a fluorescence spectrophotometer (Photon Technology International, Inc., Birmingham, AL, USA) using an excitation wavelength of λ = 295 nm, a slit width of 2.5 nm, sample temperature of 20.0 °C, and the emission spectrum at λ = 300–360 nm was analyzed. The average (*n* = 3) emission spectrum of ColoPulse-placebo was subtracted from the emission spectrum of the ColoPulse-IFX tablets (Table 2) to correct for the effects of the medium and tablet excipients during analysis.

### 2.12. ELISA Analysis

The TNF-binding potency of IFX after compounding was investigated with ELISA. A commercially available and validated ELISA kit (MabTrack M2920, Sanquin, Amsterdam, The Netherlands) was used for the analysis. This was a sandwich-type ELISA kit and contained a TNF-coated plate. Since this ELISA kit was intended for the analysis of IFX in human serum or plasma, filtered samples were first 500× diluted in human serum and this solution was thereafter further diluted 200× with the provided high performance ELISA (HPE) dilution buffer from the ELISA kit. The supplied standardized ELISA protocol of Sanquin was followed [58], which consisted of three incubation steps, two wash steps, and the addition of a stop solution upon completion of the experiment after which the OD450 nm was measured (BioTek, Winooski, VT, USA). For each experiment, quality control (QC) samples (5 mg fresh IFX stock) were analyzed as well to determine the recovery. The IFX content of all the samples was calculated with the supplied 6-point calibration curve and the content of ColoPulse-IFX was calculated with the QC values taken as 100%.

### 2.13. Comparison of the Infliximab and Caffeine Release Profiles

Caffeine was used as a model drug during the GISS experiments to investigate the in vitro ColoPulse coating performance with UV-VIS analysis (see also Section 2.14). Caffeine (25 mg) was added to the Uncoated-IFX formulation, resulting in the Uncoated-IFX-caff formulation (Table 2). The IFX release profile from Uncoated-IFX and the release profile of caffeine from Uncoated-IFX-caff was analyzed in an United States Pharmacopoeia (USP) dissolution apparatus II (Sotax, Basel, Switzerland). The dissolution medium consisted of the simulated ileum medium that was also used during the GISS [59]. The medium volume was 940 mL during the caffeine analysis, which is the same volume used during the GISS. The medium volume during the IFX analysis was 200 mL due to the lower limit of quantification of the analysis method. The medium temperature and paddle speed were 37 °C and 50 rpm, respectively. Caffeine was analyzed with UV-VIS (Thermo Fisher, Madison, WI, USA) at a wavelength of λ = 274 nm (path length of 10.00 mm). For the IFX analysis, 500 µL samples were subtracted from the medium and analyzed with the same HPLC-SEC method as described in Section 2.10.

### 2.14. Gastrointestinal Simulation System

The GISS was used to investigate the in vitro coating performance of the ColoPulse-coated tablets and to test for ileo-colonic drug targeting. The GISS is an in vitro model simulating GI transit trough the stomach (pH 1.2 for 2 h), jejunum (pH 6.8 for 2 h), ileum (pH 7.5 for 30 min), and colon (pH 6.0 for as long as needed) and is described in detail elsewhere [59]. Caffeine was added to ColoPulse-IFX (ColoPulse-IFX-caff; Table 2) as a model drug to investigate the coating performance in the GISS. This was done since the IFX release profile could not be analyzed accurately in the GISS with UV-VIS or HPLC-SEC due to the low concentration (5%–100% of the IFX dose released corresponded to 0.00025–0.0050 mg/mL). However, the addition of caffeine, which could be analyzed accurately with UV-VIS, made it possible to determine the initial moment of coating disintegration and therefore the targeted region of the formulation. An USP dissolution apparatus II (Sotax, Basel, Switzerland) coupled with an UV-VIS spectrophotometer (Thermo Fisher, Madison, WI, USA) was used during the GISS experiments. The medium temperature, paddle speed, path length, and wavelength were 37 °C, 50 rpm, 10.00 mm, and λ = 274 nm, respectively. Medium components and volume were variable since buffers were added consecutively for the pH change. The initial medium volume was 500 mL (stomach) and the final volume was 1000 mL (colon). Before and during the experiments, the pH was measured and adjusted if needed to ensure the right pH.

### 2.15. Preliminary Stability Study

ColoPulse-IFX tablets and IFX-I powder from batch VAL1 was packed in polypropylene containers and stored at 25 °C/60% RH according to ICH guidelines for 6 months [54]. The IFX-I powder and ColoPulse-IFX tablets were analyzed with the HPLC-SEC and fluorescence spectroscopy methods described in Section 2.10 and Section 2.11, respectively. ColoPulse-IFX tablets were also analyzed with the ELISA method described in Section 2.12.

## 3. Results

### 3.1. IFX-Sugar Glass Compacts

Different IFX-sugar glass compacts were produced to investigate the effects of different compaction forces on the IFX recovery and to investigate whether IFX could be released from the sugar glass matrix without the addition of tablet excipients. Table 3 shows the IFX recovery from the different sugar glass compacts compared to non-compacted IFX-sugar glass powder. Gently pulverizing non-compacted IFX-sugar glass powder did not have an effect on IFX recovery. Compacting IFX-D powder showed a noticeable decrease in IFX recovery. This effect was found acceptable when IFX-I or IFX-T was compacted at the different compaction forces (recoveries ≥ 90%). However, during the compaction experiments, it became apparent that compacting IFX-T induced crystallization of the powder. This was detrimental to IFX content (data not shown) as well as compounding due to the loss of powder flowability. Therefore, IFX-I was chosen as the optimal IFX-sugar glass powder for further compounding and the production of three consecutive validation batches of tablets (VAL1, VAL2, and VAL3, respectively). These validation batches were further analyzed.

### 3.2. Resistance to Crushing and Tablet Friability

Table 2 shows the different formulations produced during this study. The average (*n* = 5) crushing strength (range) of Uncoated-IFX tablets of VAL1, VAL2, and VAL3 were respectively 175 N (168–188 N), 178 N (174–181 N), and 174 N (164–177 N). After coating the Uncoated-IFX formulation, which resulted in the ColoPulse-IFX formulation, the average (*n* = 5) crushing strength of the validation batches increased noticeably (range 350–375 N). The Uncoated-IFX as well as ColoPulse-IFX tablets of VAL1, VAL2, and VAL3 all complied with the Ph. Eur. Friability Test (<1% loss of tablet mass) [55]. These results indicated that tablet integrity of the Uncoated-IFX and ColoPulse-IFX was ensured.

### 3.3. HPLC-SEC Analysis

**Figure 1:**
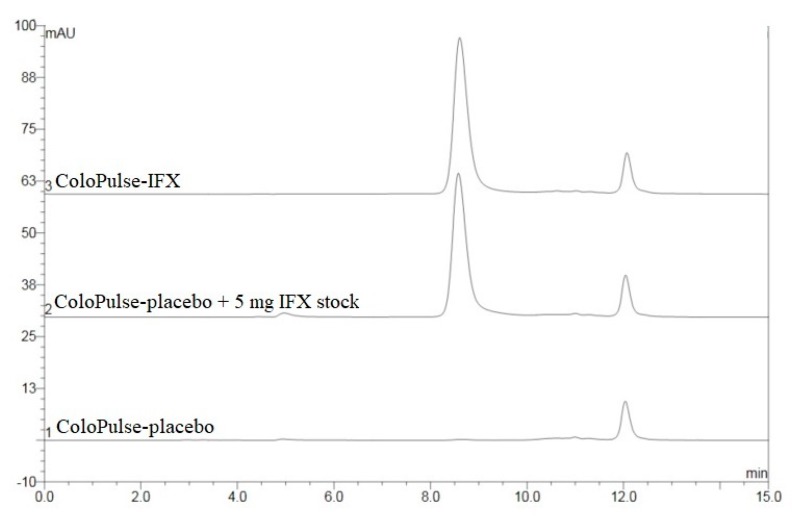
Representative HPLC-SEC chromatograms of ColoPulse-IFX, ColoPulse-placebo, and ColoPulse-placebo spiked with 5 mg fresh IFX stock.

Table 4 summarizes the results of the HPLC-SEC analyses of VAL1, VAL2, and VAL3. The average IFX content of IFX-I ranged between 88 and 91 mg IFX per gram of IFX-I powder. The theoretical content was 90 mg IFX per gram of IFX-I powder, which indicated complete recovery and fast dissolution of the IFX-I powder. The average IFX content of Uncoated-IFX and ColoPulse-IFX ranged between 94% and 101% and between 96% and 97%, respectively. The calculated acceptance value for the Content Uniformity ranged from 6.9 to 8.3 for the three validation batches, which complied with the Ph. Eur. requirement (acceptance value ≤ 15) [56]. No aggregates, fragments, or deviations were observed in the chromatograms of IFX-I and Uncoated-IFX. However, a noticeable peak in the chromatograms of the ColoPulse-IFX tablets was visible at 12 min. Analysis of ColoPulse-placebo tablets showed the same peak (Figure 1). Upon further analysis, this additional peak was attributed to Eudragit S100, which is present in the ColoPulse coating (data not shown). The results from the stress test (1 h at 60 °C) showed a substantial decrease in IFX content without the formation of additional peaks (data not shown). These results indicated that lyophilizing IFX in the inulin solution and compounding it to ColoPulse-IFX did not result in the formation of aggregates, fragments, or loss of content.

### 3.4. UV-VIS Spectroscopy Analysis

**Figure 2:**
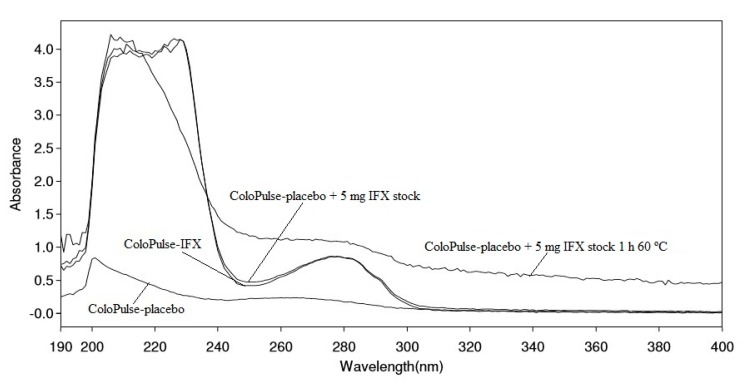
Representative UV-VIS spectra of ColoPulse-IFX, ColoPulse-placebo, ColoPulse-placebo spiked with 5 mg fresh IFX stock, and ColoPulse-placebo spiked with 5 mg fresh IFX stock stressed for 1 h at 60 °C.

During the UV-VIS spectroscopy analyses it became apparent that the IFX content in ColoPulse-IFX could not be quantified accurately due to substantial interference of the tablet excipients at λ = 280 nm. Further analysis showed that the interference was the result of the dissolved Eudragit S100, which is present in the ColoPulse coating (data not shown). Therefore, only the shape of the spectra (*n* = 3 per batch) was analyzed and compared to ColoPulse-placebo spiked with 5 mg fresh IFX stock. Figure 2 shows representative UV-VIS spectra of ColoPulse-IFX, ColoPulse-placebo, ColoPulse-placebo spiked with 5 mg fresh IFX stock, and ColoPulse-placebo spiked with 5 mg fresh IFX stock stressed for 1 h at 60 °C. The UV-VIS spectrum of ColoPulse-placebo shows the interference of the tablet excipients at λ = 280 nm. The UV-VIS spectra of ColoPulse-IFX and ColoPulse-placebo spiked with 5 mg fresh IFX stock were similar, whereas the spectrum of ColoPulse-placebo spiked with 5 mg fresh IFX stock stressed for 1 h at 60 °C showed an apparent deviation. These results indicated that lyophilizing IFX in the inulin solution and compounding it to ColoPulse-IFX did not result in apparent deviations in the UV-VIS spectrum compared to fresh IFX stock.

### 3.5. Fluorescence Spectroscopy Analysis

**Figure 3:**
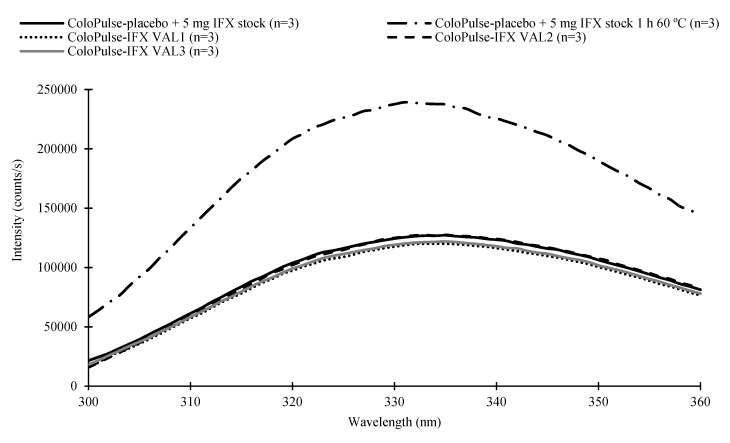
The average (*n* = 3) intrinsic fluorescence spectra of the three validation batches (VAL1-3) of ColoPulse-IFX, ColoPulse-placebo spiked with 5 mg fresh IFX stock, and ColoPulse-placebo spiked with 5 mg fresh IFX stock stressed for 1 h at 60 °C. Excitation wavelength was λ = 295 nm.

Figure 3 shows the intrinsic fluorescence spectra of ColoPulse-IFX from all three validation batches as well as the spectra of ColoPulse-placebo spiked with 5 mg fresh IFX stock and ColoPulse-placebo spiked with 5 mg fresh IFX stock stressed for 1 h at 60 °C. For these experiments, an excitation wavelength of λ = 295 nm was chosen to selectively excite the amino acid tryptophan. The intrinsic fluorescence spectrum of a protein depends on several factors. The rationale behind selectively exciting tryptophan is that its fluorescence spectrum is strongly influenced by its local environment. Changes in this local environment, which may be the results of protein (un)folding and aggregation, generally result in an alteration of the fluorescence spectrum. Hence, the tertiary structure of proteins can be investigated with this technique [57]. The spectra of ColoPulse-IFX from the three validation batches did not differ noticeably from each other or from the spectrum of ColoPulse-placebo spiked with 5 mg fresh IFX. However, a noticeable increase in fluorescence intensity was observed for ColoPulse-placebo spiked with 5 mg fresh IFX stock stressed for 1 h at 60 °C, indicating alterations in the tertiary structure of IFX. This was likely the results of protein denaturation. The results indicated that lyophilizing IFX in the inulin solution and compounding it to ColoPulse-IFX did not result in apparent alterations of the tertiary structure of IFX analyzed with fluorescence spectroscopy.

### 3.6. ELISA Analysis

**Figure 4:**
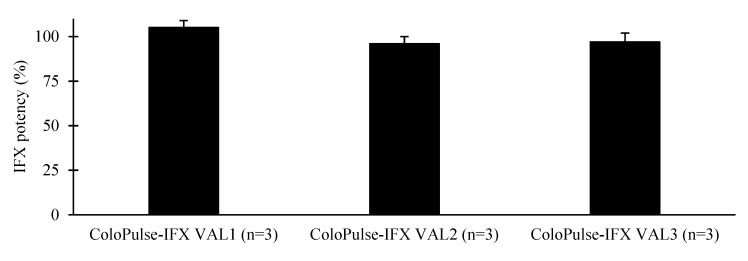
The average ± SD (*n* = 3) potency of IFX present in ColoPulse-IFX compared to 5 mg fresh IFX stock (100%) as analyzed with a validated ELISA kit.

Figure 4 shows the results of the ELISA analyses of the three validation batches. IFX in the ColoPulse-IFX tablets from VAL1, VAL2, and VAL 3 was able to bind TNF with respective average ± SD (*n* = 3) potencies of 105 ± 4%, 96 ± 4%, and 97 ± 5%, compared to fresh IFX stock. These results indicated that the compounded IFX in the ColoPulse-IFX was able to bind TNF with a potency equivalent to fresh IFX stock with no remarkable loss of antigenicity.

### 3.7. Comparison of the Infliximab and Caffeine Release Profiles

The release profile of IFX could not be analyzed accurately in the GISS due to the low concentration (5%–100% of the IFX dose released corresponded to 0.00025–0.0050 mg/mL; see also Section 2.14). Therefore, caffeine was added as model drug since this could be analyzed accurately with UV-VIS. It was not expected that the addition of 25 mg caffeine altered the formulation characteristics in a substantial manner. Since caffeine was a model drug, the release profile of caffeine in Uncoated-IFX-caff and the release profile of IFX in Uncoated-IFX were first compared in the simulated ileum medium of the GISS, which was the target site for drug release. This was done in order to investigate whether these release profiles were comparable.

**Figure 5:**
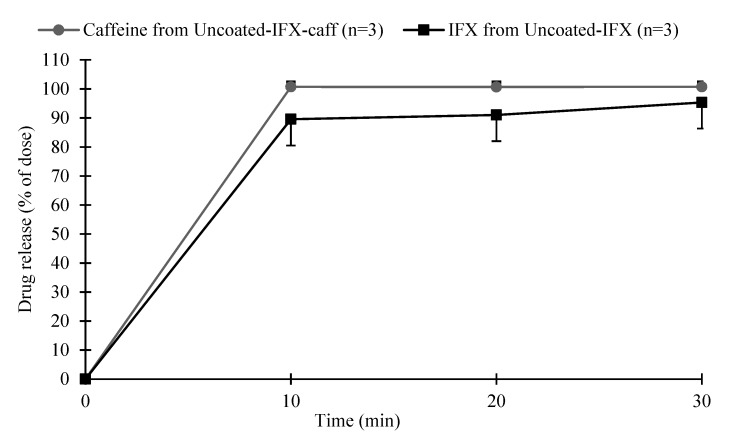
The average ± SD IFX (*n* = 3) and caffeine (*n* = 3) release profiles from Uncoated-IFX and Uncoated-IFX-caff, respectively, in the simulated ileum medium (pH 7.5) of the GISS.

Figure 5 shows the IFX and caffeine release profiles of Uncoated-IFX and Uncoated-IFX-caff, respectively, in the simulated ileum medium of the GISS. Both IFX and caffeine were released in an immediate manner. The average ± SD (*n* = 3) IFX release from Uncoated-IFX at 10 min, 20 min, and 30 min was 90 ± 9%, 91 ± 9%, and 95 ± 9%, respectively, and complied with the drug release requirement set in Table 1. Caffeine release (average ± SD) from Uncoated-IFX-caff (*n* = 3) was complete at 10 min (101 ± 2%). Although caffeine release was faster, both release profiles were considered comparable in the simulated ileum medium of the GISS with regards to complete drug release at 30 min. Therefore, caffeine release correlated with IFX release from the tablet core in the simulated ileum medium of the GISS.

### 3.8. Gastrointestinal Simulation System

**Figure 6:**
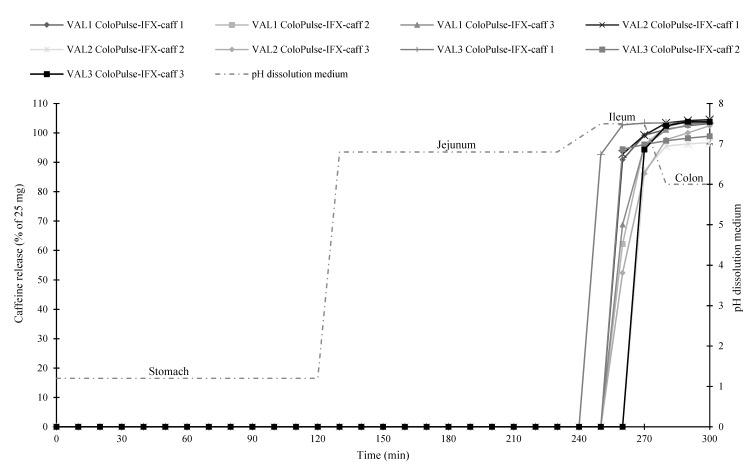
The results of the gastrointestinal simulation system (GISS) of the three validation batches (VAL1-3). Caffeine (25 mg) was added to ColoPulse-IFX (ColoPulse-IFX-caff) to investigate the in vitro coating performance with UV-VIS spectroscopy (see also Section 2.14).

In order to investigate the in vitro coating performance of ColoPulse-IFX, caffeine was added as a model drug (ColoPulse-IFX-caff) to analyze the target site of the formulation (see also Section 3.7). Figure 5 shows that caffeine release correlated with IFX release from the tablet core in the simulated ileum medium of the GISS. Figure 6 shows the results of the GISS experiments of the three validation batches. All tested ColoPulse-IFX-caff (*n* = 3 per batch) tablets complied with the requirement (Table 1) and were targeted to the simulated ileo-colonic region since no caffeine release was observed before the simulated ileum, whereas the initial site of caffeine release was in the simulated ileum. The caffeine was released immediately from the ColoPulse-IFX-caff tablets, indicating fast coating as well as tablet core disintegration in the GISS. Although IFX concentrations were not measured directly (see also Section 3.7), it is expected that IFX was released in an immediate manner comparable with the release profile of caffeine since the tablet core disintegrated rapidly and completely in the simulated ileum of the GISS (see also Figure 5). These results indicated that the ColoPulse-IFX-caff formulation was targeted to the simulated ileo-colonic region and that the tablet core formulation did not affect the in vitro ColoPulse coating performance.

### 3.9. Preliminary Stability Study

Table 5 shows the results of the preliminary stability study. No remarkable decrease of IFX content in the IFX-I powder, analyzed with HPLC-SEC, was observed at 6 months. Furthermore, no remarkable decrease of IFX content and potency in ColoPulse-IFX, analyzed with HPLC-SEC and ELISA respectively, was observed at 6 months. The HPLC-SEC chromatograms and fluorescence spectra of IFX-I and ColoPulse-IFX showed no apparent deviations compared to fresh IFX stock (data not shown). Therefore, at 6 months, ColoPulse-IFX complied with the content and potency requirements set in Table 1.

## 4. Discussion

The ColoPulse-IFX tablet is a novel formulation intended for the oral treatment of ileo-colonic IBD. Analyses of IFX content, tertiary protein structure, potentially formed aggregates, and potency showed that the IFX present in ColoPulse-IFX was stable during compounding and could bind to TNF with an equivalent potency as fresh IFX stock. Furthermore, ColoPulse-IFX was targeted to the simulated ileo-colonic region and the formulation was stable for up to 6 months stored at room temperature. In addition, the formulation complied with the Friability and Content Uniformity requirements stated in the Ph. Eur. monographs, which are requisite for oral dosage forms intended for human use. Thus, we present a feasible and validated production process of ileo-colonic-targeted IFX tablets for the oral treatment of ileo-colonic IBD.

The powder of Remsima vials, which already contained the disaccharide sucrose, was lyophilized with different sugars for additional stability of the IFX. The stability of proteins in dry-powder formulations can be enhanced by incorporating the protein in a sugar glass matrix. This enhanced stability is generally thought to be the result of protein structure preservation in a rigid, amorphous sugar glass matrix by means of water replacement and vitrification, limiting molecular mobility and thereby protein degradation [50].

Further enhancement of protein stability in dry-powder formulations can be achieved by applying a combination of sugars with different molecular characteristics, such as size and molecular flexibility, with the objective of combining the stabilizing characteristics of both sugars in one matrix [53]. Hence, we investigated three sugar candidates with different molecular characteristics for the additional stabilization of IFX. It was first investigated whether IFX was readily released from the compacts of the lyophilized sugar glass powder without tablet excipients, since only then an incomplete IFX release could be attributed to the compacted sugar glass matrix characteristics and not, for instance, to the adsorption to insoluble tablet excipients of the tablet core. IFX was not readily released from the dextran sugar glass compacts. Compaction of the trehalose sugar glass powder resulted in crystallization of the matrix, which was detrimental to the IFX content. Crystallization of trehalose compacts has been described elsewhere and may be the result of the applied compaction force and moisture content [60]. Compacting as well as gently pulverizing IFX-I had no remarkable effect on the IFX recovery, and therefore, this formulation was chosen for further compounding.

The stresses associated with compounding IFX-I to ColoPulse-IFX were not detrimental to the IFX structure and potency, making the production of 5 mg ColoPulse-IFX tablets feasible. Both IFX-I and ColoPulse-IFX were stable for up to 6 months stored at room temperature according to the ICH guideline [54]. Storage at room temperature was opted in view of convenience and patient-friendliness. However, long-term protein stability may be compromised when protein formulations are stored at room temperature. Alternatively, protein formulations may be stored refrigerated in view of increasing the long-term stability. A disadvantage, however, is the humid environment of refrigerated storage. Water, which may penetrate into the formulation, can act as a plasticizer in sugar glass matrices and may lower the *T*_g_ of the sugar glass matrix. If the *T*_g_ is reached, the matrix transitions from a rigid glassy state to a mobile rubbery state. This transition may be detrimental to protein stability, since the vitrification is lost. The increased mobility due to this transition may also result in alterations in the protein structure, and therefore, loss of activity [50]. Hence, both storage conditions have advantages and disadvantages. At the time of writing, we initiated a long-term stability study in which we investigate the stability of ColoPulse-IFX as well as IFX-I for two years stored at room temperature and refrigerated according to ICH guidelines [54].

We opted for a 5 mg IFX dose based on the intravenously administered dose, which generally is 5 mg/kg given every 2 to 8 weeks. The frequency of administration depends on the induction or maintenance phase. For example, a patient weighing 75 kg would normally receive a dose of 375 mg every 8 weeks during the maintenance phase. This would correspond to a daily dose of 6.7 mg. Tablets containing 5 mg IFX result in convenient daily dosage regimens in view of the oral treatment of IBD calculated based on bodyweight. In the mentioned example, the patient might receive 5 mg ColoPulse-IFX once (rounded down) or twice (rounded up) daily.

The release profile of IFX in the simulated ileum medium of the GISS showed that IFX was released immediately and completely from the tablet core. Furthermore, the release profiles of IFX and the model drug caffeine were comparable. It is therefore expected that the IFX dose is released completely (≥85%) at the targeted site. From a pharmacokinetic and pharmacodynamic point of view, an intravenously administered dose that exerts its effect systemically for weeks cannot be converted to a daily oral dosage intended for topical treatment. There is, however, no other feasible and rational manner to determine the oral dosage of IFX at this moment. We therefore think that our approach is a starting point in a dose-finding study.

Excluding extraintestinal manifestations of disease activity, the inflammation in ileo-colonic IBD is localized and restricted to the terminal ileum and colon. Intravenously administered IFX is highly efficacious [16,19]. However, there are several disadvantages associated with this route of administration. First, patients need to visit a hospital, and trained personnel need to prepare the IFX infusions. Second, intravenous administration exposes the patient systemically to IFX, and thus, antagonizes TNF systemically rather than only locally at the site of inflammation. Systemic IFX exposure is associated with an increased risk of opportunistic infections and the development of ATI, which can result in infusion reactions and the loss of response to IFX therapy [20,22,23]. These disadvantages may be eliminated if IFX were to be administered orally. In addition, daily oral treatment with IFX continuously exposes the ileo-colonic region to relatively high concentrations of IFX since the entire 5 mg dose per administration is released at the site of inflammation without the substantial dilution and gradual clearance of the dose, which does occur following intravenous administration.

Studies indeed show that the anti-inflammatory effects of IFX result, at least partly, from local immunomodulation at the sites of inflammation and that the IFX concentration at these sites correlates with the clinical response instead of serum concentration alone [25,26,27,29,30]. Interestingly, treatment of active UC with AVX-470—an orally administered, bovine-derived, topically active anti-TNF drug—in a randomized, double-blind, placebo-controlled trial showed a reduction in TNF as well as inflammatory biomarkers in colonic tissue. This was associated with a clinical response in a dose-dependent manner [34,35]. In addition, studies have shown that local administration of IFX in symptomatic isolated intestinal lesions [31] and postoperative recurrent [33], fistulizing [61,62,63,64], or stricturing CD [32,65] ameliorates symptoms and could be an effective treatment option for patients not responding to conventional therapy. For some patients, locally administered IFX induced complete remission, even after a follow-up period of 14–32 months [31,33]. No serious adverse events were observed during these studies and one study reported no ATI development after a 6 month follow-up period [64]. Furthermore, patients were included that did not respond to or had a contra-indication for systemically administered IFX and showed a clinical response to locally administered IFX [31,61,63,64,65]. Altogether, these results suggest that active inflammation in IBD can indeed be treated with local TNF inhibition.

The environment of the GIT is hostile for proteins due to the initial low pH of the stomach and the presence of proteolytic enzymes, such as pepsin, trypsin, chymotrypsin, carboxypeptidase, and elastase [39,66,67]. Interestingly enough, studies show a recovery of up to 50% of immunologically active immunoglobulins—a protein structure similar to IFX—in feces after oral administration and transit through the entire GIT. These studies report no substantial systemic absorption of the immunoglobulins [45,46]. Even though immunoglobulins were partly digested during GI transit, activity could still be observed when the antigen-binding fragments (Fab) stayed intact. Applying an enteric coating to the immunoglobulin formulation enhanced the recovery in feces. It is therefore expected that a greater fraction of orally administered proteins remain intact and active when targeted to the ileo-colonic region since the average pH in this region is relatively neutral (pH 6–8) and the presence of proteolytic enzymes is less abundant compared to the small intestines [36,45,46,68]. Although bacterial proteases present in the colon may degrade proteins, immunoglobulin coating of fecal bacteria has been reported in IBD patients as well as healthy individuals. Loss of IFX into feces by ulcerated epithelial surfaces in UC patients has also been reported. The recovered IFX in the feces, which transited (partly) through the colon, was still active and could bind to TNF. Taken together, these results show that antibodies and IFX remain, at least partly, intact and active during colonic transit [47,69,70,71].

In vitro results investigating the stability of IFX in simulated GI fluids show that IFX is not stable in gastric conditions due to the low pH instead of pepsin activity [48]. The stability in the simulated small intestines was better and could be increased by the addition of proteins, which simulated competition of food proteins with IFX for proteolytic degradation [49]. IFX stability in simulated human colonic conditions, which contained fecal bacteria as well, was however the greatest. Intact IFX recovery after 1 h and 2 h in these conditions was respectively 75% and 40%, and a great fraction of the digested IFX was fragmented into Fab and F(ab′)_2_ fragments, which may be able to still neutralize TNF [45,46,48].

The strength of this study was the utilization of different analytic techniques used in protein analysis to investigate the structural integrity and potency of IFX in ColoPulse-IFX compared to fresh IFX stock. Additionally, in vitro ileo-colonic targeting of this formulation was investigated and achieved by the ColoPulse coating technology. We have previously shown in five clinical trials that this coating targets the ileo-colonic region in healthy subjects as well as CD patients [38,40,41,42,43]. However, a limitation of this present study is that all these in vitro experiments give limited insight into the in vivo formulation and IFX characteristics in IBD patients. For instance, it is currently unknown what the mucosal IFX concentration and stability at the targeted site will be. Furthermore, the pharmacokinetics of ColoPulse-IFX and ATI development during oral treatment remains to be investigated. These questions should be addressed in a clinical trial. Moreover, the long-term stability (12–24 months) of this formulation should also be investigated. This long-term stability study is initiated at the time of writing. Parallel to this, we are preparing a clinical trial to investigate the efficacy and applicability of ColoPulse-IFX in ileo-colonic IBD.

## 5. Conclusions

In conclusion, ColoPulse-IFX is an ileo-colonic-targeted tablet intended for the oral and topical treatment of IBD. The production process of this formulation was validated, and analyses showed that the structural integrity and potency of IFX in ColoPulse-IFX was equivalent to fresh IFX stock. The formulation was stable for up to 6 months stored at room temperature. Therefore, ColoPulse-IFX is an interesting novel formulation for the oral treatment of ileo-colonic IBD. This should be investigated in a clinical trial.

## 6. Patents

The ColoPulse coating technology is patented [72].

## Figures and Tables

**Table 1:** Target product profile of the ileo-colonic-targeted infliximab (IFX) tablets (ColoPulse-IFX).

Parameter	Requirement
Tablet shape	Biconvex, round, 9 mm
Uncoated tablet mass	350 mg
Content ^a^	90%–110% of claim
IFX release ^a^	≥85% of the dose within 30 min in simulated ileum
Tertiary protein structure ^b^	No apparent deviations compared to fresh IFX stock
Soluble aggregates/fragments ^a^	No apparent deviations compared to fresh IFX stock
IFX potency ^c^	90%–110% compared to fresh IFX stock
Applied coating ^d^	11–15 mg/cm^2^
Ileo-colonic targeting ^e^	Initial coating disintegration in the simulated ileum

^a^: Determined with HPLC-SEC. ^b^: Determined with fluorescence spectroscopy. ^c^: Determined with ELISA. ^d^: Expressed as mg Eudragit S100 per cm^2^ of tablet surface. ^e^: Determined in a gastrointestinal in vitro model (GISS).

**Table 2:** The components of the different formulations. Abbreviation: IFX-I: Infliximab reconstituted with the inulin solution and thereafter lyophilized.

Components	Uncoated-IFX	Uncoated-IFX-Caff	ColoPulse-IFX	ColoPulse-IFX-Caff	ColoPulse-Placebo
IFX-I ^a^	5 mg IFX	5 mg IFX	5 mg IFX	5 mg IFX	-
Inulin (mg)	-	-	-	-	55 ^b^
Microcrystalline cellulose (mg)	Ad 350	Ad 350	Ad 350	Ad 350	Ad 350
Silicon dioxide (mg)	3.5	3.5	3.5	3.5	3.5
Croscarmellose sodium (mg)	14	14	14	14	14
Sodium stearyl fumarate (mg)	3.5	3.5	3.5	3.5	3.5
Caffeine (mg)	-	25	-	25	-
ColoPulse (mg/cm^2^) ^c^	-	-	11–15	11–15	11–15

^a^: Amount varied as the content of IFX per gram IFX-I differed from batch to batch. Range: 88–91 mg IFX per gram IFX-I. Theoretical IFX content was 90 mg IFX per gram IFX-I. ^b^: Amount corresponding to the average amount of added IFX-I in the formulations containing IFX. ^c^: Amount of coating is expressed as applied Eudragit S100 per cm^2^ of tablet surface.

**Table 3:** The average ± SD (*n* = 2) IFX recovery from the different IFX-sugar glass compacts. Abbreviations: Comp. 1 kN: IFX-sugar glass powder compacted at 1 kN. Comp. 3 kN: IFX-sugar glass powder compacted at 3 kN. IFX-D: infliximab-dextran sugar compact. IFX-I: infliximab-inulin sugar compact. IFX-T: infliximab-trehalose sugar compact. Non-comp.: non-compacted IFX-sugar glass powder. Non-comp. pulv.: non-compacted IFX-sugar glass powder pulverized in a mortar with a pestle.

Sugar	Processing ^a^	Content (IFX mg/g) ^b^	Recovery (%) ^b^
IFX-I	Non-comp.	87 ± 0.1	97 ± 0.1
	Non-comp. pulv.	89 ± 2	99 ± 2
	Comp. 1 kN	82 ± 0.2	91 ± 0.2
	Comp. 3 kN	82 ± 0.7	91 ± 0.8
IFX-D	Non-comp.	82 ± 0.3	91 ± 0.3
	Non-comp. pulv.	80 ± 0.1	89 ± 0.1
	Comp. 1 kN	73 ± 0.3	81 ± 0.3
	Comp. 3 kN	69 ± 1	77 ± 1
IFX-T	Non-comp.	83 ± 0.1	92 ± 0.1
	Non-comp. pulv.	82 ± 0.3	91 ± 0.3
	Comp. 1 kN	82 ± 0.6	91 ± 0.7
	Comp. 3 kN	80 ± 1	89 ± 1

^a^: Non-compacted IFX-sugar glass powder was pulverized to mimic pulverization of the compacts and to investigate whether this process had an effect on content recovery. ^b^: Theoretical content was 90 mg IFX per gram of IFX-sugar glass powder.

**Table 4:** The results of the HPLC-SEC analyses of the validation batches (VAL1-3). IFX-I (*n* = 3 for each batch) was analyzed to determine the amount of IFX-I needed for 5 mg IFX tablets. IFX content is expressed as percentage of 5 mg for the Uncoated-IFX (*n* = 3 for each batch) and ColoPulse-IFX (*n* = 10 for each batch) tablets. The acceptance value was calculated according to the Ph. Eur. [56].

Analysis	VAL1	VAL2	VAL3
IFX-I (mg/g) ^a^	91 ± 1	88 ± 0.5	89 ± 2
Uncoated-IFX (%)	101 ± 2	94 ± 1	96 ± 3
ColoPulse-IFX (%)	96 ± 2	97 ± 3	96 ± 2
Acceptance Value ^b^	6.9	8.3	7.3
Aggregates/fragments	Not observable	Not observable	Not observable

^a^: Theoretical content was 90 mg IFX per gram of IFX-I powder. ^b^: Calculated for ColoPulse-IFX. Ph. Eur. requirement is ≤15.0 [56].

**Table 5:** The average ± SD preliminary stability data of IFX-I (*n* = 3) and ColoPulse-IFX (*n* = 3) stored in polypropylene containers at 25 °C/60% RH conform ICH guidelines [54].

IFX-I (mg IFX/g Powder)		ColoPulse-IFX (% of 5 mg)			
0 months	6 months	0 months		6 months	
HPLC-SEC	HPLC-SEC	ELISA	HPLC-SEC	ELISA	HPLC-SEC
91 ± 1.0	92 ± 0.4	105 ± 4	97 ± 2	98 ± 2	103 ± 1
